# Mechanism Underlying Triple VEGFR Inhibitor Tivozanib-Induced Hypertension in Mice Model

**DOI:** 10.3390/ph16020295

**Published:** 2023-02-14

**Authors:** Wael A. Alanazi, Abdulrahman S. Alanazi, Doaa M. El-Nagar, Abdullah M. Aljuraybah, Sary Alsanea, Metab Alharbi

**Affiliations:** 1 Department of Pharmacology and Toxicology, College of Pharmacy, King Saud University, Riyadh 11451, Saudi Arabia; 2 Department of Zoology, College of Science, King Saud University, Riyadh 11451, Saudi Arabia

**Keywords:** tivozanib, VEGFR, angiotensin-II (AngII), endothelin-1 (ET-1), oxidative stress, nitric oxide (NO)

## Abstract

Tivozanib is a triple vascular endothelial growth factor receptor inhibitor, recently approved for the treatment of refractory advanced renal cell carcinoma. Clinical studies showed that around 46% of patients who received tivozanib suffer from hypertension in all grades. Thus, the present study was conducted to identify the role of angiotensin-II (AngII) in the mechanism underlying tivozanib-induced vascular toxicity and hypertension. C57BL/6 male mice received tivozanib (1 mg/kg) with or without losartan (10 or 30 mg/kg) for 3 weeks. Blood pressure was recorded every 3 days, and proteinuria was measured every week. On day 21, all mice were euthanized, and samples were harvested for further analysis. Tivozanib elevated blood pressure until systolic blood pressure reached 163 ± 6.6 mmHg on day 21 of treatment with low urination and high proteinuria. AngII and its receptors, endothelin-1, and oxidative stress markers were significantly increased. While nitric oxide (NO) levels were reduced in plasma and aortic tissues. AngII type 1 receptor blockade by losartan prevented these consequences caused by tivozanib and kept blood pressure within normal range. The results showed that AngII and ET-1 might be potential targets in the clinical studies and management of hypertension induced by tivozanib.

## 1. Introduction

According to the American College of Cardiology/American Heart Association (ACC/AHA), the risk factors of hypertension (HTN) can be genetic predisposition and environmental factors such as obesity and sodium intake [1]. Blood pressure is determined by various cardiovascular system characteristics, including blood volume and cardiac output, as well as the balance of arterial tone, which is influenced by both intravascular volume and neurohumoral systems [1].

Angiotensin-II (AngII) is a potent vasoconstrictor hormone that has a crucial role in maintaining the hemodynamic function, glomerular filtration rate (GFR) and glomerular perfusion pressure [2,3]. AngII binds to the type I (AT1R) and type II (AT2R) angiotensin receptors, with the AT1R receptor being the best characterized [4]. AT1R was targeted by losartan, which was the first angiotensin-II receptor blocker (ARB) to be approved for the treatment of hypertension in the late 1990s, and it was quickly followed by candesartan, irbesartan, valsartan, telmisartan and olmesartan [5]. All ARBs have a high affinity for the AT1R, which is abundant in different organs, including smooth muscle cells, the heart, kidneys and the aorta [6]. They are commonly used in clinical practice to treat hypertension, congestive heart failure and nephropathies [6]. Therapeutic AT1R antagonists, often known as “sartans,” have been developed and are presently being therapies to counteract the AT1R-mediated cardiovascular and renal effects of AngII [5,6].

Dysregulated RTKs have been linked to the development of cancer over the past two decades, making them a target for tyrosine kinase inhibitors (TKIs) [7,8]. Despite the clinical advantages of TKIs, the toxicity and organ dysfunction they produce lower the quality of life and the commitment to therapy [9]. Cardiotoxicity, hypertension, heart hypertrophy and myocardial infarction are the main side effects of TKI therapy that vary in frequency and severity amongst TKIs [10]. Recently, the resistance of tumors against vascular endothelial growth factor receptor (VEGFR) inhibitors have been associated with the crosstalk between tumor angiogenesis and immune cells [11]. Thus, treatment approaches, including multiple VEGFR inhibitors for the prevention of tumor resistance, may increase the incidence of side effects of VEGFR inhibitors. 

Tivozanib is an oral VEGFR-TKI specific for VEGFR1-3. It was approved by the United States Food and Drug Administration (FDA) in March 2021 for the treatment of adult patients with relapsed or refractory advanced renal cell carcinoma (RCC) [12]. Induction of hypertension and proteinuria are two of the main adverse effects of tivozanib [12]. Tivozanib has the potential to produce severe hypertension and a hypertensive crisis [13]. Hypertension occurred in 46% of tivozanib-treated patients, with 22% of the events ≥ Grade 3, a hypertensive crisis occurred in 0.8% of those treated after administering an overdose of tivozanib and one patient (0.1%) died from a hypertensive emergence [13]. Proteinuria was reported in 8% of tivozanib-treated patients [14]. Furthermore, tivozanib can result in myocardial ischemia and arterial thromboembolic events that are significant, even deadly [15]. In patients receiving tivozanib, cardiac ischemia occurred in 3.2% of cases, with 1.5% of events being ≥ grade 3 and 0.4% of events being fatal [15]. Patients receiving tivozanib reported 2% of arterial thromboembolic events, including 0.1% of ischemic stroke-related deaths [15]. Still, there is a lack of investigations about the mechanism underlying tivozanib-induced hypertension and its correlation with the renin angiotensin-II system (RAS). No prospective study identified the role of AngII in the induction of hypertension and renal cardiovascular damage. Thus, the current study was conducted to investigate the role of tivozanib in the activation of AngII/AT1R/ET-1 causing oxidative stress and reduction of nitric oxide bioavailability leading to hypertension. Also, this study evaluated AT1R blockade via losartan in attenuation of consequences of tivozanib treatment on blood pressure.

## 2. Results

### 2.1. Effect of Tivozanib and/or Losartan on the Survival Probability and Body Weight of the Mice

**Figure 1:**
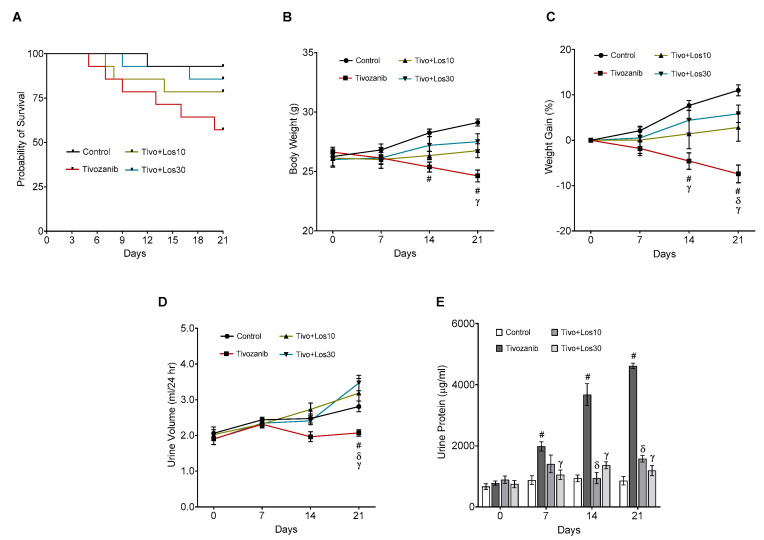
Effect of tivozanib and losartan on survival rate, body weight, urine flow and urine protein levels. Mice either daily received tivozanib (1 mg/kg), tivozanib plus losartan (10 mg/kg; Tivo + Los10), tivozanib plus losartan (30 mg/kg; Tivo + Los30) or vehicle control. (**A**) Probability of survival. (**B**) Body weight measurement every 7 days. (**C**) Weight gain percentage was calculated using the body weight measurement. (**D**) Urine volume was measured weekly using metabolic cages. (**E**) Urine protein concentration. Values are expressed as the mean ± SEM (*n*= 6–8). ^#^ *p* < 0.05 (Control vs. Tivozanib). ^δ^ *p* < 0.05 (Tivozanib vs. Tivo + Los10). ᵞ *p* < 0.05 (Tivozanib vs. Tivo + Los30).

Tivozanib reduced the survival rate, body weight and weight gain percentage (%) in mice treated with tivozanib alone as compared to the control group (Figure 1A–C). The combination of tivozanib and losartan at doses 10 and 30 mg/kg increased the survival rate of mice as compared with the tivozanib alone treated group (Figure 1A). On days 14 and 21, the body weight and weight gain % were significantly improved in the combination treatment in comparison with the tivozanib alone group (Figure 1B,C).

### 2.2. Effect of Tivozanib and/or Losartan on the Urine Flow and Levels of Urine Protein

Tivozanib significantly reduced urine flow after 3 weeks of treatment and increased proteinuria on days 7, 14 and 21 as compared with the control group (Figure 1D,E). In contrast, concomitant treatment of losartan with tivozanib notably regulated the urine flow and decreased proteinuria as compared with the tivozanib alone group (Figure 1D,E).

### 2.3. Effect of Tivozanib and/or Losartan on the Hemodynamic Function and Morphological Changes of the Aorta

**Figure 2:**
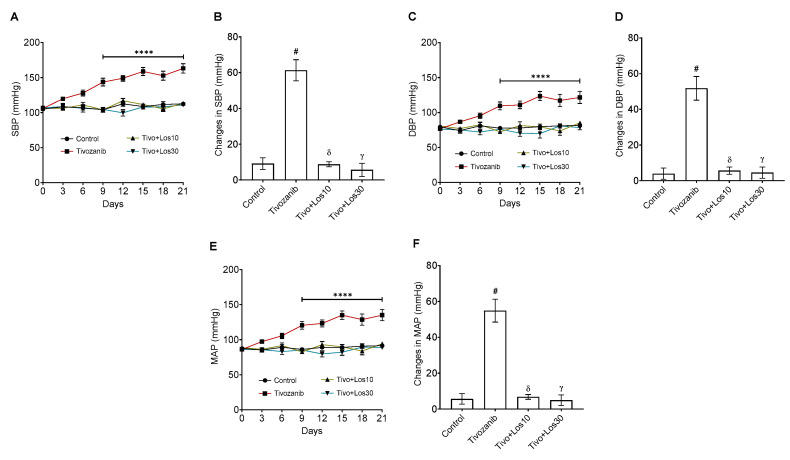
Effect of tivozanib and losartan on blood pressure and morphological changes to the aorta. Mice either daily received tivozanib (1 mg/kg), tivozanib plus losartan (10 mg/kg; Tivo + Los10), tivozanib plus losartan (30 mg/kg; Tivo + Los30) or vehicle control. (**A**) Systolic blood pressure (SBP). (**C**) Diastolic blood pressure (DBP). (**E**) Mean arterial pressure (MAP). (**B**,**D**,**F**) Changes in SBP, DBP and MAP were calculated by taking the differences between baseline and data on day 21, respectively. Values are expressed as the mean ± SEM (*n* = 8). ^****^p < 0.0001 (Control vs. Tivozanib). ^#^ *p* < 0.0001 (Control vs. Tivozanib). ᵟ *p* < 0.0001 (Tivozanib vs. Tivo + Los10). ᵞ *p* < 0.0001 (Tivozanib vs. Tivo + Los30).

On the hemodynamic levels, the blood pressure parameters such as systolic blood pressure (SBP), diastolic blood pressure (DBP) and mean arterial pressure (MAP) were recorded every three days until the end of the treatment. The results showed that tivozanib increased all these parameters on day 3, and the data showed a significant elevation in SBP, DBP and MAP from day 6 (127 ± 4.2 mmHg, 95.4 ± 3.9 mmHg, 105.5 ± 3.8 mmHg) until day 21 (163 ± 6.6 mmHg, 121.6 ± 8.7 mmHg, 135 ± 7.9 mmHg) as compared with the baseline (day 0; 106 ± 1.5 mmHg, 76.8 ± 1.7 mmHg, 86 ± 1.6 mmHg, respectively; Figure 2A,C,E). In comparison with the control group, tivozanib significantly increased all blood pressure parameters (SBP, DBP and MAP; Figure 2A,C,E). Also, the changes in these parameters were elevated in the tivozanib group as compared with the control group (Figure 2B,D,F). In contrast, concomitant treatment of losartan with tivozanib controlled the blood pressure at both doses of losartan (10 and 30 mg/kg) and being within the normal range during the study as compared with the tivozanib alone group (Figure 2A–F). In addition, the heart rate was recorded, but there was no significant change among all treated groups (data not shown). 

**Figure 3:**
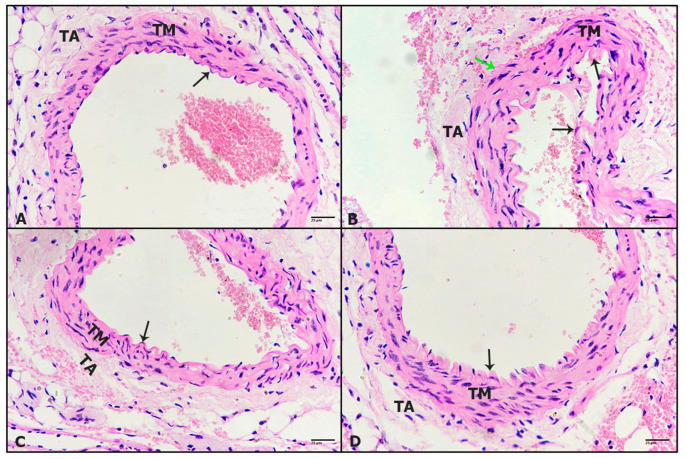
Effect of tivozanib and losartan on histology of heart and kidney. Mice either daily received tivozanib (1 mg/kg), tivozanib plus losartan (10 mg/kg; Tivo + Los10), tivozanib plus losartan (30 mg/kg; Tivo + Los30) or vehicle control. Photomicrographs of mice aorta. (**A**) Untreated control showing normal aorta structure. (**B**) Aorta treated with tivozanib revealing destructed tunica intima and the shrinkage of elastic fibers. (**C**) Aorta treated with Tivo + Los10 displaying improved structure. (**D**) Aorta treated with Tivo + Los30 showing healthier aortic layers. Hematoxylin and eosin at 400× magnification (H&E-400X).

On the histological levels, the dorsal aorta in control mice showed normal appearance and layers, tunica intima, tunica media and tunica adventitia (Figure 3A). The aorta of mice treated with tivozanib displayed intense constriction resulting in the destruction of the tunica intima, a narrow, collapsed lumen and the shrinkage of elastic fibers at the edge of tunica media (Figure 3B). Mice aorta treated with tivozanib and losartan (10 mg/kg; Tivo + Los10) revealed improvement without collapsing (Figure 3C). Moreover, mice aorta treated with tivozanib and losartan (30 mg/kg; Tivo + Los30) posted healthy opened aorta with improved layers (Figure 3D).

### 2.4. Effect of Tivozanib and/or Losartan on Cardiac and Renal Histopathology

**Figure 4:**
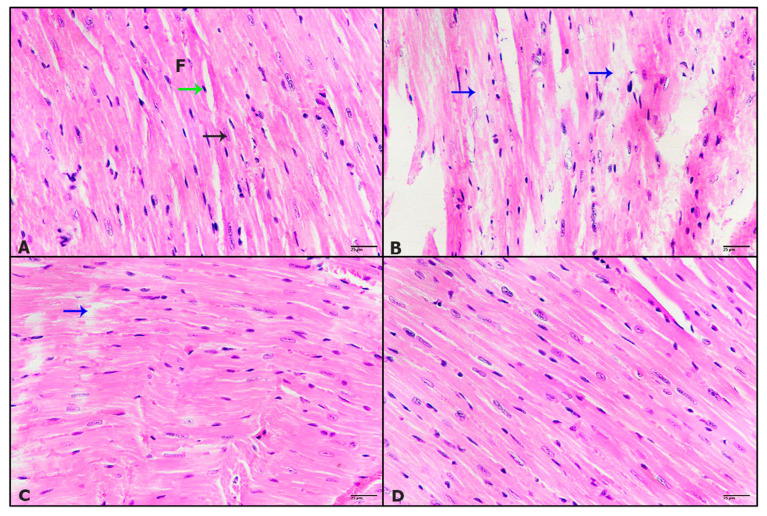
Effect of tivozanib and losartan on histology of heart and kidney. Mice either daily received tivozanib (1 mg/kg), tivozanib plus losartan (10 mg/kg; Tivo + Los10), tivozanib plus losartan (30 mg/kg; Tivo + Los30) or vehicle control. Photomicrographs of mice cardiac tissue. (**A**) Untreated control showing normal feature myocardial fiber, central nucleus (black arrow) and endothelial nucleus (green arrow). (**B**) Mice treated with tivozanib revealing severe myocardial degeneration (blue arrows). (**C**) Mice treated with Tivo + Los10 displaying improvement with slight myocardial degeneration (blue arrow). (**D**) Mice treated with Tivo + Los30 showing more improvement. Hematoxylin and eosin at 400× magnification (H&E-400X).

The histological studies showed that untreated control cardiac tissue has normal histological features, as the myocardial fibers looked healthy with central disc nuclei and the endothelial nuclei at the edge of the fibers (Figure 4A). Meanwhile, mice treated with tivozanib displayed severe degeneration of myocardial fibers (Figure 4B). Mice aorta treated with tivozanib and losartan (10 mg/kg; Tivo + Los10) revealed marked improvement except for some myocardial fibers degeneration (Figure 4C). Furtherly, increasing the dose of losartan to reach 30 mg/kg (Tivo + Los30) posted more improvement that cardiac tissue looked healthy as untreated control (Figure 4D). 

**Figure 5:**
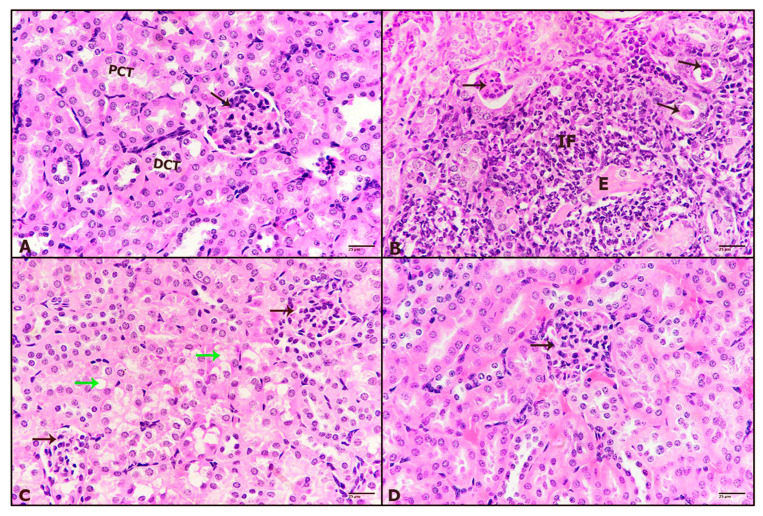
Effect of tivozanib and losartan on histology of heart and kidney. Mice either daily received tivozanib (1 mg/kg), tivozanib plus losartan (10 mg/kg; Tivo + Los10), tivozanib plus losartan (30 mg/kg; Tivo + Los30) or vehicle control. Photomicrographs of mice renal tissue. (**A**) Untreated control revealing normal appearance. (**B**) Mice treated with tivozanib exhibiting granulomatous inflammation (IF) and edema (E). (**C**) Mice treated with Tivo + Los10 showed tubular vacuolar degeneration (green arrows). (**D**) Mice treated with Tivo + Los30 showing marked improvement. (Black arrows) glomeruli, (PCT) proximal convoluted tubules and (DCT) distal convoluted tubules. Hematoxylin and eosin at 400× magnification (H&E-400X).

In the kidneys, control mice kidney revealed normal structure with abundant glomerulus in between tubules inside the cortex area (Figure 5A). While mice treated with tivozanib exhibited a massive incidence of granulomatous inflammation and edema, glomeruli showed severe atrophy (Figure 5B). Otherwise, mice treated with Tivo + Los10 displayed noticeable improvement in the glomerulus, but tubules revealed marked vacuolar degeneration (Figure 5C). Additionally, mice treated with Tivo + los30 showed great improvement in glomeruli and tubules (Figure 5D).

### 2.5. Effect of Tivozanib and/or Losartan on Production of Angiotensin-II and AT1/AT2 Receptors Expression

**Figure 6:**
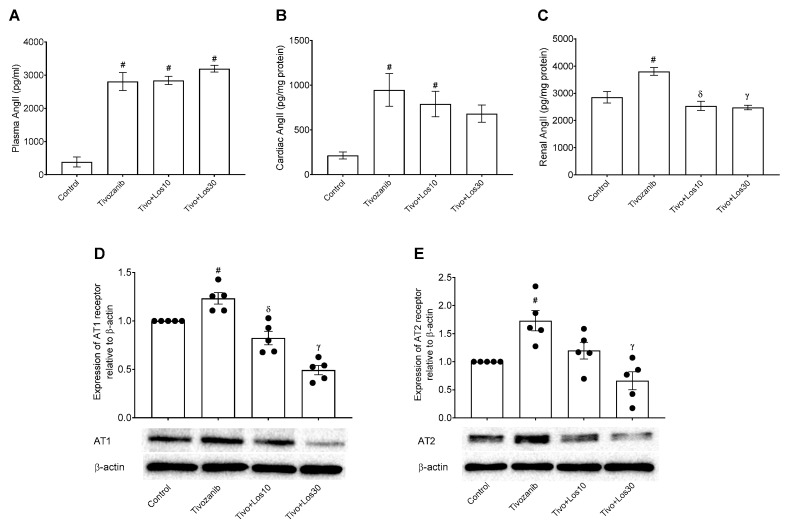
Effect of tivozanib and losartan on angiotensin-II levels and AT1/AT2 receptors expression in the aorta. Mice either daily received tivozanib (1 mg/kg), tivozanib plus losartan (10 mg/kg; Tivo + Los10), tivozanib plus losartan (30 mg/kg; Tivo + Los30) or vehicle control. (**A**) Plasma levels of angiotensin-II (AngII). (**B**) AngII levels in hearts. (**C**) AngII levels in kidneys. (**D**) Protein expression of angiotensin-II type 1 (AT1) receptor in the aorta. (E) Protein expression of angiotensin-II type 2 (AT2) receptor in the aorta. Values are expressed as the mean ± SEM (*n* = 5). ^#^ *p* < 0.05 (Control vs. Tivozanib; Control vs. Tivo + Los10), (Control vs. Tivo + Los30). ^δ^ *p* < 0.001 (Tivozanib vs. Tivo + Los10). ᵞ *p* < 0.001 (Tivozanib vs. Tivo + Los30).

The current study investigated the mechanism underlying tivozanib-induced hypertension by assessing the role of AngII via blocking its AT1R using losartan during tivozanib treatment. Thus, AngII levels were measured, and the results showed a significant increase in AngII in the plasma, heart and kidneys of mice treated with tivozanib alone as compared with the control group (Figure 6A–C). In plasma and hearts, concomitant treatment of losartan with tivozanib didn’t change the mediated AngII by tivozanib, whereas tivozanib plus losartan at a dose of 10 mg/kg showed a significant increase in comparison with the control group (Figure 6A,B). In contrast, losartan treatment (10 and 30 mg/kg) significantly decreased AngII induced by tivozanib in the Tivo + los10 and Tivo + Los30 groups compared with the tivozanib alone group (Figure 6C). For further investigation, AngII receptor (AT1R and AT2R) expression was assessed in aortic tissues using western blotting analysis. As expected, both receptors were highly expressed in the tivozanib group and decreased in the combination treatment (Tivo + Los30; Figure 6D,E). In Tivo + Los10 group, AT1R and AT2R expression was downregulated by losartan, but it was a significant inhibition in AT1R expression as compared with the tivozanib group (Figure 6D,E). In contrast, increasing the dose of losartan to 30 mg/kg showed significant inhibition of both receptors (Figure 6D,E).

### 2.6. Effect of Tivozanib and/or Losartan on Production of Endothelin-1 and Nitric Oxide

**Figure 7:**
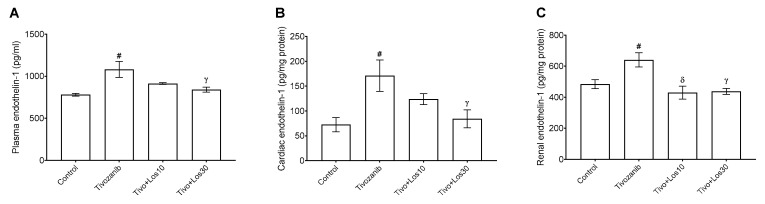
Effect of tivozanib and losartan on endothelin-1 levels. Mice either daily received tivozanib (1 mg/kg), tivozanib plus losartan (10 mg/kg; Tivo + Los10), tivozanib plus losartan (30 mg/kg; Tivo + Los30) or vehicle control. (**A**) Plasma levels of endothelin-1. (**B**) Endothelin-1 levels in hearts. (**C**) Endothelin-1 levels in kidneys. Values are expressed as the mean ± SEM (*n* = 5). ^#^ *p* < 0.05 (Control vs. Tivozanib). ᵟ *p* < 0.01 (Tivozanib vs. Tivo + Los10). ᵞ *p* < 0.05 (Tivozanib vs. Tivo + Los30).

**Figure 8:**
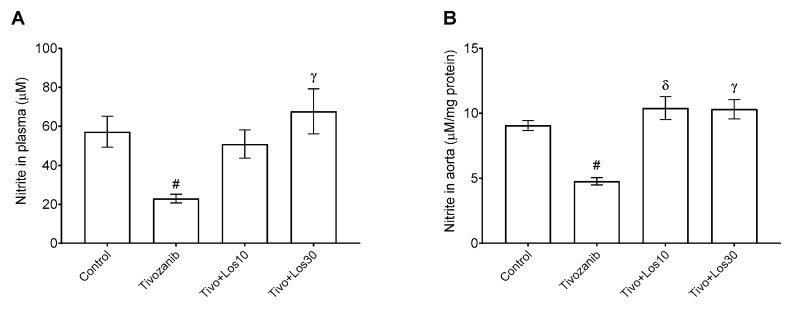
Effect of tivozanib and losartan on nitrite levels. Mice either daily received tivozanib (1 mg/kg), tivozanib plus losartan (10 mg/kg; Tivo + Los10), tivozanib plus losartan (30 mg/kg; Tivo + Los30) or vehicle control. (**A**) Nitrite levels in plasma. (**B**) Nitrite levels in the aorta. Values are expressed as the mean ± SEM (*n* = 5–6). ^#^ *p* < 0.05 (Control vs. Tivozanib). ^δ^ *p* < 0.001 (Tivozanib vs. Tivo + Los10). ᵞ *p* < 0.01 (Tivozanib vs. Tivo + Los30).

The current study assessed the level of ET-1, which is well-known as a potent vasoconstrictor. Our results showed that ET-1 levels were increased in plasma, heart and kidney during tivozanib alone treatment (Figure 7A–C). Losartan (30 mg/kg) significantly inhibited ET-1 induced by tivozanib in the Tivo + Los30 group to reach normal levels in plasma, heart and kidney (Figure 7A–C). Nitric oxide (NO) levels were measured indirectly through the determination of nitrite (NO_2_) concentration as an oxidized form of NO. In both plasma and aorta, NO_2_ levels were decreased in the tivozanib alone group (Figure 8A,B). In the Tivo + Los10 group, the NO_2_ concentration was significantly increased in the aorta and elevated in plasma but was not significantly increased compared with the tivozanib alone treated group (Figure 8A,B). In contrast, losartan (30 mg/kg) increased NO_2_ significantly in both plasma and the aorta in the Tivo + Los30 group (Figure 8A,B).

### 2.7. Effect of Tivozanib and/or Losartan on Levels of Oxidative Stress Marker in Aorta, Heart and Kidney

**Figure 9:**
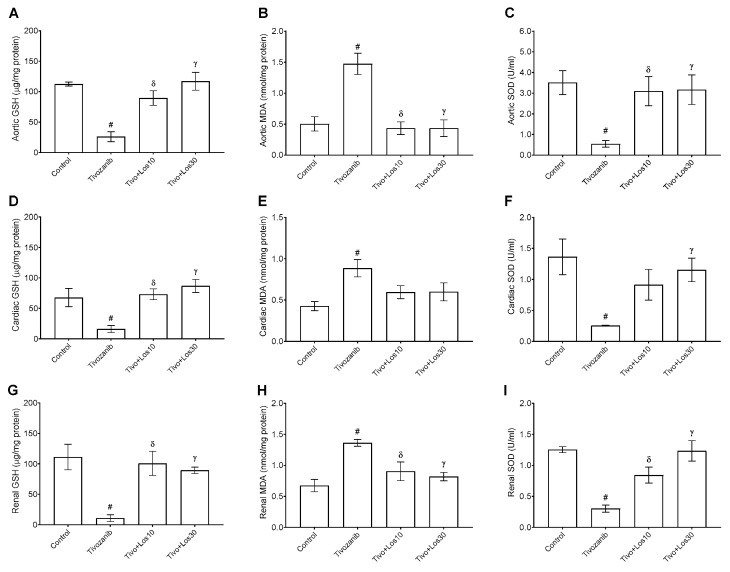
Effect of tivozanib and losartan on oxidative stress markers including glutathione (GSH), malondialdehyde (MDA) and superoxide dismutase (SOD). Mice either daily received tivozanib (1 mg/kg), tivozanib plus losartan (10 mg/kg; Tivo + Los10), tivozanib plus losartan (30 mg/kg; Tivo + Los30) or vehicle control. (**A**–**C**) GSH, MDA and SOD levels in aortic tissues. (**D**–**F**) GSH, MDA, SOD levels in cardiac tissues. (**G**–**I**) GSH, MDA and SOD levels in renal tissues, respectively. Values are expressed as the mean ± SEM (*n* = 5–6). ^#^ *p* < 0.05 (Control vs. Tivozanib). ᵟ *p* < 0.05 (Tivozanib vs. Tivo + Los10). ᵞ *p* < 0.05 (Tivozanib vs. Tivo + Los30).

Tivozanib has been implicated in the induction of oxidative stress. Thus, the present study assessed the effects of concomitant treatment of losartan with tivozanib on oxidative stress via measurement of oxidative stress markers, including glutathione (GSH), malondialdehyde (MDA), superoxide dismutase (SOD). The antioxidants markers (GSH and SOD) were reduced in the aortic, cardiac and renal tissues in the tivozanib group and returned to the normal levels by losartan (10 and 30 mg/kg) in the Tivo + Los10 and Tivo + Los30 groups, respectively (Figure 9A–I). In contrast, the lipid peroxidation marker (MDA) was increased by tivozanib and reduced by losartan treatment in the aorta, heart and kidney (Figure 9A–I). However, AT1R blockade via losartan showed that AngII/AT1R activation is the main activator of tivozanib-induced oxidative stress.

## 3. Discussion

In cancer patients treated with tivozanib, clinical trials showed that around 45% of them suffered from hypertension as the most common adverse reaction, and 8% were reported with proteinuria [13,14,16]. Still, the causes of tivozanib-induced hypertension are not clearly identified. Thus, the current study was conducted to study the role of tivozanib in the induction of hypertension through the activation of the AngII/AT1R signaling pathway leading to increased ET-1 and reduced NO bioavailability in the vasculatures. The results revealed that tivozanib treatment-induced vascular toxicity and hypertension could be manifested in different ways, including increasing blood pressure (SBP = 163 mmHg), reduction of urine volume and increasing proteinuria. In the vasculature, results indicated that tivozanib caused morphological changes in the aorta and caused vasoconstriction-induced hypertension. In addition, the mortality rate was higher among mice treated with tivozanib alone, which might be due to the inhibition of VEGF receptors 1, 2 and 3. While blocking of AT1R via losartan prevented these consequences of tivozanib treatment. At the molecular level, tivozanib increased plasma AngII and ET-1 formation, and AT1R was overexpressed in the aortic tissues. These indicated factors have been implicated in the reduction of NO bioavailability and oxidative stress leading to endothelial injury and hypertension, as shown in the current study and consistent with previous studies [17,18,19]. Losartan treatment attenuated the overexpression of AT1 and AT2 receptors in aortic tissues leading to regulation of AngII-induced ET-1 production, oxidative stress and improved NO content in the vasculature. However, losartan regulated the hemodynamic function during tivozanib treatment. On the histological levels, losartan mitigated the vascular toxicity of tivozanib and showed a healthy opened aorta with improved layers. These findings are consistent with the recent recommendations of using angiotensin system inhibitors, including angiotensin-converting enzyme inhibitors (ACEs) and angiotensin-II type 1 receptor blockers (ARBs) as a therapy in the management of hypertension caused by VEGFR-TKIs including sorafenib, sunitinib, tivozanib [16,20]. Although RAAS is crucially involved in the pathogenesis of essential hypertension, there is an absence of evidence that RAAS contributes significantly to hypertension caused by VEGFR-TKI [20]. A previous study revealed that sunitinib treatment elevated the circulatory levels of ET-1, leading to endothelial dysfunction and hypertension in the treated subjects [21]. In addition, in several preclinical and clinical studies, VEGFR-TKIs have been linked to an increase in circulation ET-1 levels, which is correlated with the severity of VEGFR-induced hypertension [22,23,24]. A recent study demonstrated that sunitinib-induced hypertension could be managed by ET-1 receptor blockers in rats [25]. Still, the correlation between RAAS and VEGFR-TKI-induced hypertension is not clearly identified [26]. 

Abundant evidence showed the role of losartan in the prevention of oxidative stress in several pathological conditions-caused hypertension, such as hypoxia, diabetes and cardiomyopathy [27,28,29]. In addition, losartan metabolites have shown a beneficial antioxidant effect via inhibition of nicotinamide adenine dinucleotide phosphate oxidase (NADPH)-induced free radical formation [30]. The aortic content of antioxidants (GSH and SOD) and oxidant (MDA as a lipid peroxidation marker) during tivozanib treatment have been evaluated for the first time in the current study, and it revealed that three weeks of tivozanib treatment dysregulated GSH, SOD and MDA levels in aortic tissues. In contrast, losartan balanced the content of oxidative stress markers in the aorta. These results are compatible with previous research studies showing that AT1R blockade via losartan ameliorated the imbalance of the redox process and normalized the aortic levels of GSH, SOD and MDA in the hypertensive subjects [31,32]. Taken together, tivozanib promoted AngII production, causing induction of ET-1 release and oxidative stress. These factors contributed to a decrease in NO bioavailability in blood vessels leading to hypertension.

In hearts and kidneys, the effect of tivozanib has been evaluated in the current study, whereas both organs have essential roles in the regulation of arterial blood pressure and proteinuria [33,34]. Three weeks of tivozanib treatment caused a reduction in urination and increasing in proteinuria. Tivozanib exaggerated the production of AngII and ET-1 in cardiac and renal tissues. Meanwhile, blocking AT1R via losartan attenuated the activity of AngII induced by tivozanib and decreased ET-1 release in both the heart and kidneys. In contrast, AngII formation induced by tivozanib was decreased in kidneys during losartan treatment, while losartan has no effect on AngII levels in the circulation and heart. These results are in accordance with earlier research that revealed that losartan attenuates intrarenal local production of AngII with no significant change in its plasma and cardiac levels [35]. As previously approved, induction of AngII and ET-1 has been implicated in causing acute and chronic kidney diseases, cardiac hypertrophy and heart failure leading to end organ damage [36,37,38,39]. In addition to the antioxidant effects of losartan in aortic tissues, it also normalized GSH, SOD and LPO levels in the renal and cardiac tissues, leading to the prevention of the systemic oxidative stress induced by tivozanib. In subjects treated with VEGFR-TKIs such as sorafenib and gefitinib, research revealed that these drugs increased free radical production and oxidative damage, which contribute to causing kidney and heart diseases [40,41,42]. Our recent study demonstrated that AngII type1 receptor blockade attenuated oxidative stress and AngII/AT1R/MAPK signaling pathway in gefitinib-induced cardiac hypertrophy [42]. Based on the current finding, the signaling pathways involved in tivozanib-induced nephrotoxicity and cardiotoxicity need further investigations to be clarified.

To our knowledge, there is no prospective study identifying the link between the recently approved triple VEGFR Inhibitor, tivozanib and AngII/ET-1/Oxidative stress/NO in the induction of hypertension and renal cardiovascular damage. Still, further studies are required to clarify the factors involved in AngII formation and the role of ET-1 and its receptors in tivozanib-induced hypertension.

## 4. Materials and Methods

### 4.1. Animals and Treatments

C57BL/6J mice (8-week-old male; *n* = 40) were obtained from the Animal Care Center, College of Pharmacy, King Saud University (Riyadh, Saudi Arabia). Mice were fed with a standard chow pellet diet and had free access to water under controlled conditions (23 ± 2 °C and a 12 h light/dark cycle). All animal experiments described in this study complied with the National Institutes of Health guidelines for the Care and Use of Laboratory Animals and were approved by the local institutional research ethics committee of King Saud University on 14 October 2021 (KSU-SE-21–61).

Mice were randomly divided into 4 groups (10 mice per group). The first group (control group) received an intraperitoneal injection (i.p.) of normal saline and an oral dose (p.o.) of dimethyl sulfoxide (DMSO < 1%) for 21 consecutive days. The second group (tivozanib group); mice in this group were treated with tivozanib (MedChemExpress, Monmouth Junction, NJ, USA; 1 mg/kg/day, p.o.) and normal saline (0.5 ml/day, i.p.) for 21 days. In the third group, mice were co-treated with losartan (Ak Scientific, Union City, CA, USA; 10 mg/kg/day, i.p.) and tivozanib (1 mg/kg/day, p.o.) for 21 days. The last group was treated with losartan (30 mg/kg/day, i.p.) and tivozanib (1 mg/kg/day, p.o.) for 21 days. Doses and durations were selected based on previous protocols [43,44,45]. Tivozanib was dissolved in DMSO < 1% and losartan in normal saline, and the final volume of all doses was 0.5 mL. During treatment, blood pressure was measured by using a non-invasive blood pressure system. Urine samples were collected by metabolic cages on days 0, 7, 14 and 21, and the body weight of mice was measured every three days. Urine samples were analyzed for measuring proteinuria using bicinchoninic acid (BCA) assay kit (Molequle-On, New Lynn, AKL, New Zealand). On day 22, mice were anesthetized using a ketamine/xylazine mixture (i.p.). Blood was collected immediately from the posterior vena cava, and the plasma was separated for biochemical analysis. Hearts, kidneys and aorta samples were harvested for histological and biochemical analysis.

### 4.2. Blood Pressure Measurement Using a Non-Invasive Tail-Cuff System

Non-invasive blood pressure system (CODA^®^ High Throughput System, Kent Scientific, Torrington, CT, USA) was used to measure four physiological hemodynamic parameters, including SBP, DBP, MAP and HR. This device inflates the cuff until the pulse disappears; then, the cuff pressure is lowered gently from the systolic blood pressure. The pulse reappears when blood pressure falls under systolic pressure [46].

### 4.3. Histopathological Analysis

Tissues were gathered from all mice groups at the time of euthanization and preserved in a 4% formaldehyde solution immediately. The fixed tissues were subsequently fixed in paraffin, and micrometer-thin sections of 3–4 µm were prepared by microtome. The organs sections were next deparaffinized and stained with hematoxylin and eosin (H&E). Under a light microscope, the degree of heart, kidneys and dorsal artery injuries was assessed for histologic examination [47].

### 4.4. Measurement of Angiotensin-II and Endothelin-1 Levels Using Enzyme-Linked Immunosorbent Assays

Enzyme-Linked Immunosorbent Assay (ELISA) was used to measure the plasma, heart and kidney of AngII and ET-1 levels (ABclonal Technology, Woburn, MA, USA) as per the manufacturer’s protocol. The collected samples (plasma and tissues homogenate) were incubated with biotin conjugate antigen working solution for 1 h at 37 °C. After washing, streptavidin-horseradish peroxidase (HRP) working solution was added to each well and incubated for 30 min at 37 °C. Then, 90 µL of tetramethylbenzidine (TMB) substrate was added to each well and incubated for 20 min at 37 °C. Stop solution (50 μL/well) was added, and the optical density of each well was determined within 5 min, using a microplate reader set to 450 nm.

### 4.5. Nitrite Determination Assay

Based on the enzymatic conversion of nitrate to nitrite (NO_2_) by nitrate reductase, NO was determined via the QuantiChrom^TM^ Nitric Oxide Assay Kit (Bioassays Systems, Hayward, CA, USA) for the colorimetric detection of total nitrite in the dorsal aorta and plasma. Aorta (80 mg) was homogenized in phosphate-buffered saline (PBS; 300 µL), and then the protein concentration in the collected supernatant was determined using a BCA assay. NO_2_ measurement was carried out according to the manufacturer’s instructions, and at the end of the procedure, the plate (96 wells) was read at 540 nm.

### 4.6. Protein Expression of Angiotensin-II Type 1 and 2 Receptors Using Western Blot Analysis

Aortic tissues were homogenized in ice-cold RIPA lysis buffer with cocktail protease inhibitor (ThermoFisher Scientific, Waltham, MA, USA). The protein concentrations were then measured using the bicinchoninic acid assay (BCA) method using a commercially available kit after the protein lysates (Molequle-On, New Lynn, AKL, New Zealand). Polyacrylamide gel electrophoresis (PAGE) was used to separate the proteins by mass and charge. The addition of denaturant sodium dodecyl sulfate (SDS) made all the proteins negatively charged and separated solely by mass as they ran from the negative to the positive electrode through the polyacrylamide gel. Then, separated proteins were transferred using a transfer buffer to polyvinylidene fluoride (PVDF; Bio-Rad, Hercules, CA, USA). Following the transfer, the membranes were blocked for one hour at room temperature with 5% non-fat dry milk in Tween-TBS (Tris-Buffered Saline) with gentle rocking. Membranes were incubated with rabbit anti-angiotensin type 1 receptor (AT1R) polyclonal antibody, rabbit anti-angiotensin type 2 receptor (AT2R) polyclonal antibody (ABclonal Technology, Woburn, MA, USA) overnight at 4 °C. Then, blots were incubated with the appropriate HRP-conjugated secondary antibody (ABclonal Technology, Woburn, MA, USA) for 1 h. Thereafter, membranes were visualized using a chemiluminescence reagent (Merck Millipore, Burlington, MA, USA) and imaged using a Bio-Rad gel-imaging system (Bio-Rad, Hercules, CA, USA). β-actin monoclonal antibody (ABclonal Technology, Woburn, MA, USA) was used as a housekeeping protein. All the primary antibodies were used in a dilution of 1:1000 [42].

### 4.7. Measurement of Oxidative Stress Markers Using Biochemical Assays

Oxidative stress markers, including GSH, MDA and SOD, were measured in the aorta, hearts and kidneys using commercial biochemical assays kits (Molequle-on, New Zealand) as per the manufacturer’s protocol. Aortic, cardiac and renal tissues (100 mg) were immersed into 1 mL of the available extraction reagent of each kit and homogenized on ice, and then centrifuged at 8000 rpm for 10 min at 4 °C. Before the test, the protein concentration of the collected supernatant of each sample was measured via BCA method. Samples were mixed with reagents I and II to measure GSH content at absorbance 412 nm. MDA levels were detected after incubating the samples with an MDA working reagent and reagent III; then, the absorbance was measured at 450 nm, 532 nm and 600 nm. While SOD levels were measured in all samples via incubation with reagents I, II, II and V, then the absorbance was measured specifically at 560 nm per the manufacturer’s protocol.

### 4.8. Statistical Analysis

The data are presented as means ± SEM, and statistical significance was defined as *p*-values <0.05. Different groups were compared by 1- and 2-way ANOVA then Tukey’s multiple comparison tests were used. The statistical analyses and figures were performed using Graph Pad Prism version 9.

## 5. Conclusions

In conclusion, the mechanism underlying triple VEGFR inhibitor tivozanib-induced vascular toxicity and hypertension was investigated in the current study. It found that tivozanib induced AngII formation leading to induction of ET-1, oxidative stress and reduction of NO release causing vasoconstriction. Oxidative stress causes endothelial nitric oxide synthase (eNOS) uncoupling, leading to NO/ROS imbalance, which causes endothelial dysfunction and hypertension. Thus, future studies are needed to investigate the effect of tivozanib-induced oxidative stress on endothelial dysfunction and nitric oxide synthases, including coupled/uncoupled eNOS and iNOS. In addition, our findings showed that tivozanib-induced AngII production in the kidney and heart caused oxidative stress and organ damage. AT1R blockade by losartan prevented the renal-cardiovascular consequences caused by tivozanib. Still, the mechanisms related to these actions need to be clarified in future studies. The limitations of the study are mainly centered on the lack of data related to nitric oxide synthases and the causes of tivozanib-induced Ang-II production. The results revealed that AngII and ET-1 might be potential targets in future clinical studies and/or the management of tivozanib-caused hypertension.

## Data Availability

Data are available within the article.
